# Case Report: Multi-Orifices Vertebral Arteriovenous Fistula With Severe Scoliosis in Neurofibromatosis Type 1: Might Be a Congenital Disease With Mesodermal Dysplasia

**DOI:** 10.3389/fneur.2021.578797

**Published:** 2021-03-17

**Authors:** Yingjin Wang, Changwei Yuan, Shengli Shen, Yang Zhang, Jiayong Zhang, Hongzhou Duan

**Affiliations:** Department of Neurosurgery, Peking University First Hospital, Beijing, China

**Keywords:** arteriovenous fistula, scoliosis, neurofibromatosis (NF1), embolization treatment, multiple orifices

## Abstract

**Background:** Vertebral arteriovenous fistula (AVF) associated with neurofibromatosis type 1 (NF-1) is a rare condition in the previous reports. However, whether vertebral AVF in NF-1 is congenital or NF-1 disease progression hasn't been clarified.

**Case Description:** We reported a 48-year-old male case of vertebral AVF simultaneously combined with thoracic scoliosis and NF-1. Preoperative CT angiography showed the AVF with multiple orifices located on the vessel wall of the vertebral artery, which was proved during the procedure of endovascular treatment. By occluding the parent vertebral artery, the AVF was finally cured. Further whole-exome sequencing identified a novel germline heterozygous point nonsense mutation, c.G397T(p.E133X), in the NF1(NM_000267) gene exon4.

**Conclusions:** From this patient, we speculate that vertebral AVF associated with NF-1 might be a congenital disease as a manifestation of mesodermal dysplasia.

## Background

Epidural arteriovenous fistula (AVF) expanding over multiple cervical vertebrae was extremely rare, and most reported cases were associated with neurofibromatosis type 1 (NF-1) (1), which is an autosomal dominant disease due to mutation on chromosome 17 and has a major impact on the nervous system, eye, skin, bone, or cardiovascular system (2). Two possible mechanisms by which an AVF might arise in patients with NF-1 were proposed in the literature, (1) vascular sequelae presented as abnormalities of connective tissue which resulted in vessel vulnerability and friability; (2) congenital with a manifestation of mesodermal dysplasia (3). However, it is difficult to determine which theory is more accurate. Herein, we report a multi-orifices vertebral AVF case with NF-1, who simultaneously suffered thoracic scoliosis and received a whole-exome sequencing. From this case, we tried to explore and illustrate the underlying mechanism.

## Case Description

### Clinical Presentation

**Figure 1:**
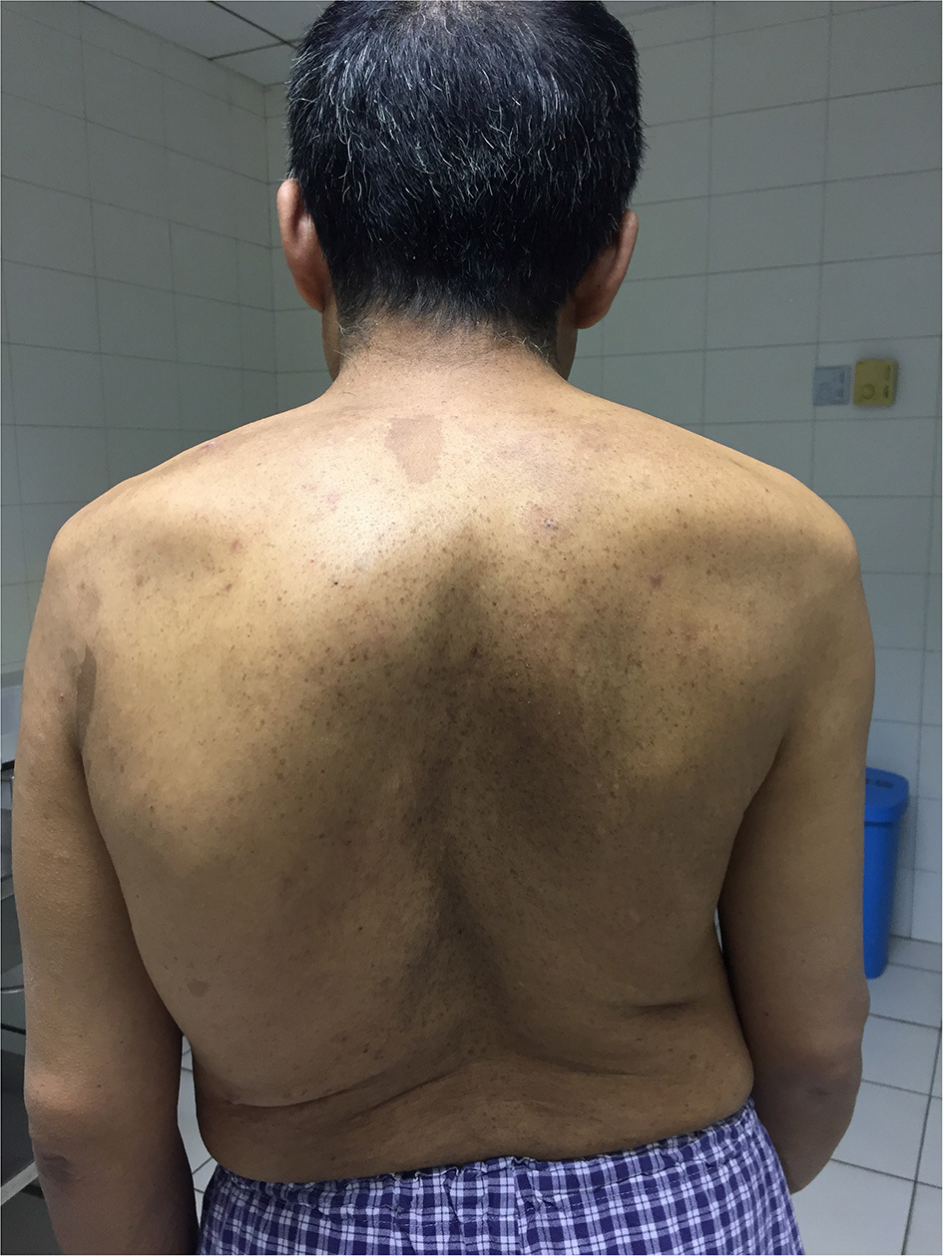
Back view of the patient showing severe thoracic scoliosis and widespread small neurofibroma and Cafe-au-Lait Spots.

A 48-year-old man with noticeable scoliosis was admitted to our hospital because of neck pain for 1 month without obvious inducement. The pain radiating to bilateral shoulders continuously existed, and occasionally it was accompanied by numbness of the left face. Other symptoms such as nausea, vomiting, dizziness, limb weakness, or defecation dysfunction were not apparent in this patient. In his history, the patient complained of left tinnitus since he was more than 10 years old which was aggravated in the latest 5 years. Head magnetic resonance imaging (MRI) and a series of examinations in otorhinolaryngology didn't find the cause of tinnitus. Thoracic scoliosis was noticed when the patient was about 20 years old and it has become more severe in the past 10 years. There was no similar case in the patient's family. Physical examination showed numerous café-au-lait spots and dermal neurofibroma together with severe thoracic scoliosis (Figure 1).

### Radiologic Findings

**Figure 2:**
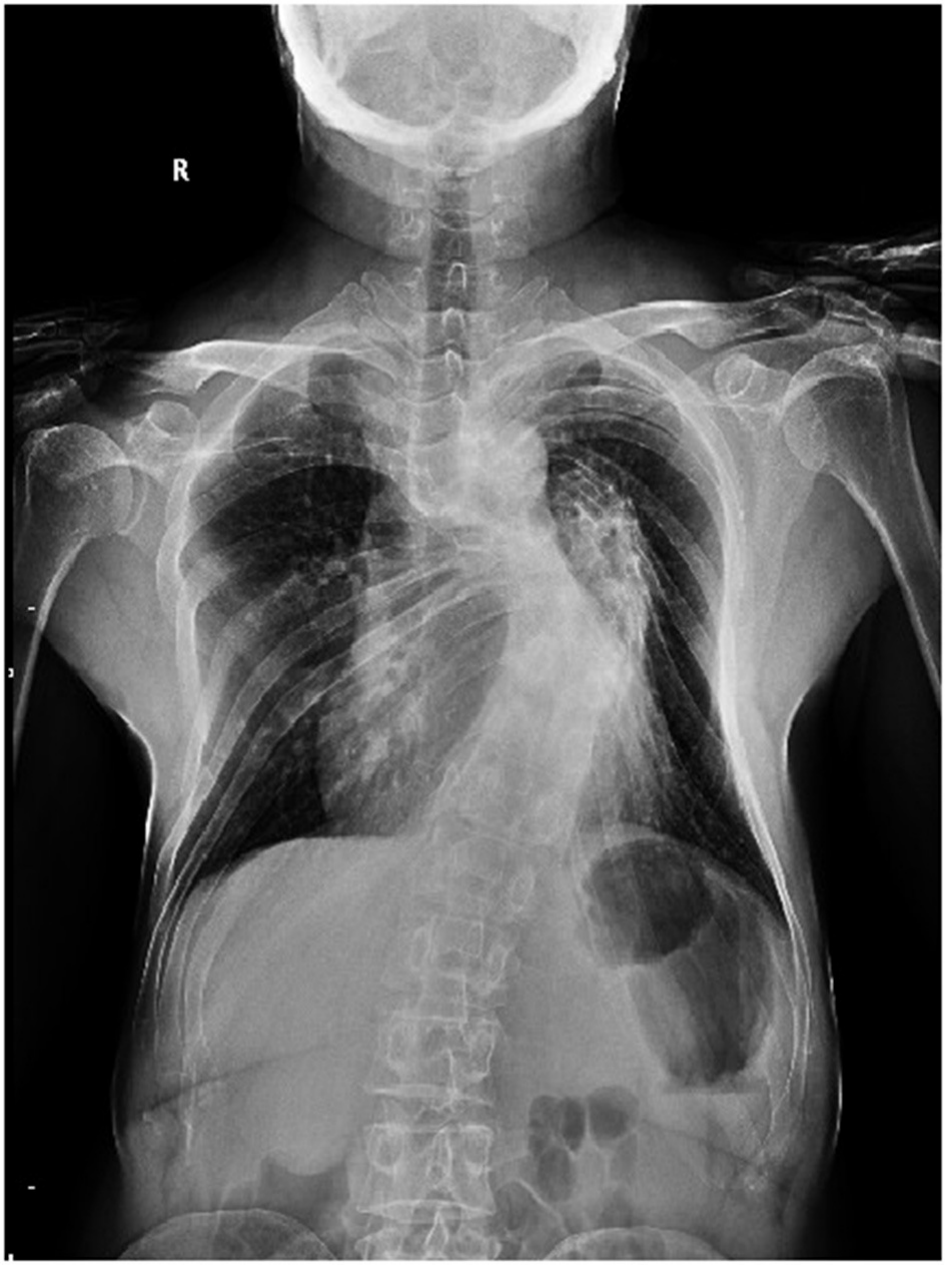
X-radiograph showing the patient with a severe thoracic scoliosis and barrel chest.

**Figure 3:**
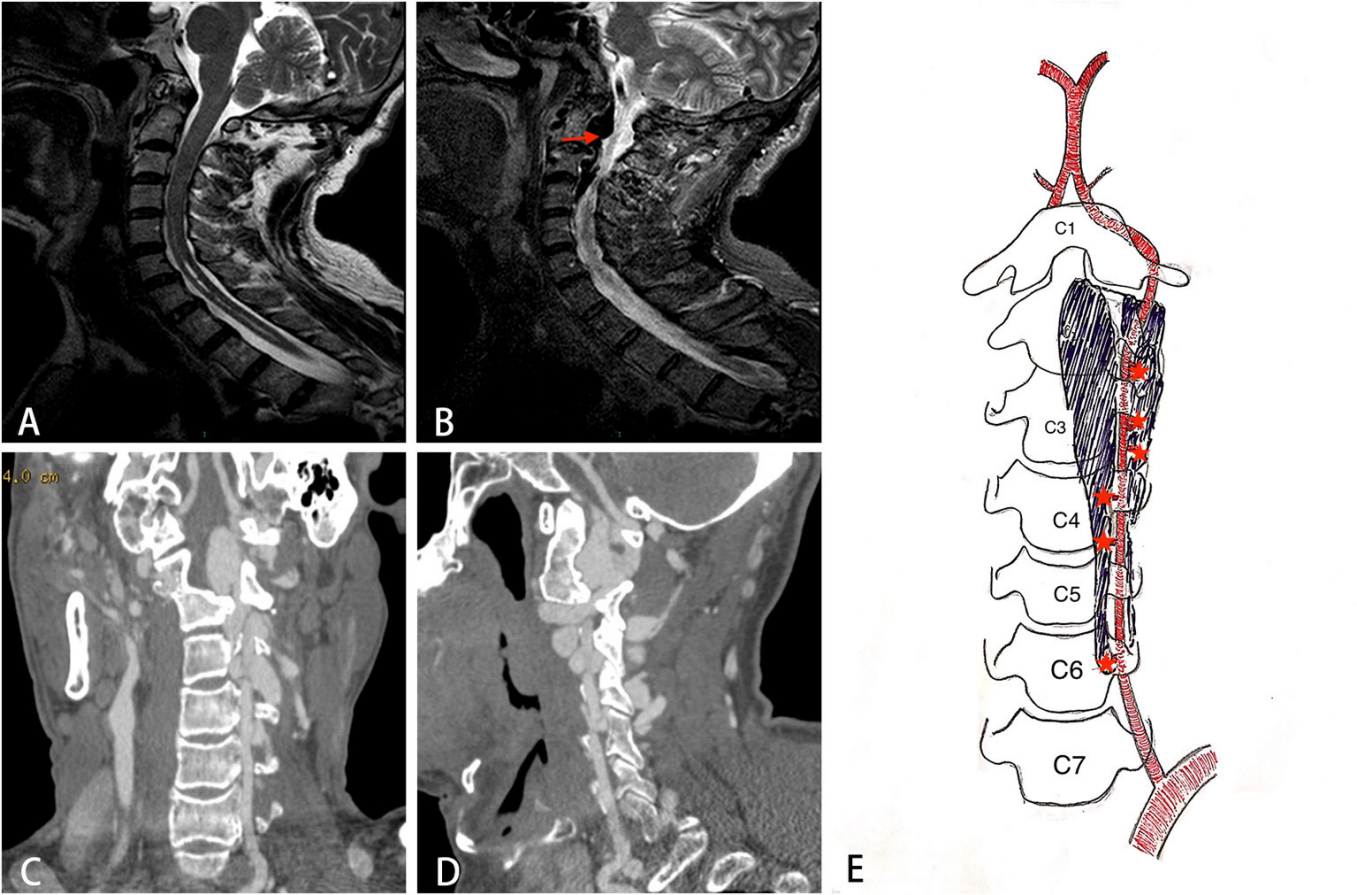
The imaging findings of the patient. **(A)** Sagittal T2 weighted image showing syringomyelia exists in the C5-T2 spinal cord. **(B)** A hypointense flow void signal lesion (arrow) extending from C2 to C4 is located in the cervical canal and front of the left cervical spine. **(C, D)** The coronal and sagittal view of CT angiography showing a complicated AVF at the level of C2-C6. **(E)** A schematic diagram of the structure of AVF. Star indicates the fistula orifices.

Chest X-ray confirmed the existence of thoracic scoliosis (Figure 2). Cervical T2-weighted MRI showed a syringomyelia existed in the spinal cord from C5 to T2 (Figure 3A) and a hypointense signal lesion extended from C2 to C4 which was located in the cervical canal and in front of the left cervical spine, compressing the cervical nerve root (Figure 3B). The flow void signal of the lesion suggested it might be a vascular lesion. Further cervical computed tomography angiography (CTA) illustrated a complicated AVF at the level of C2-C6 with enlargement of the C2-C4 intravertebral foramen (Figures 3C,D). From the original images of CTA, multiple fistula orifices could be identified (Figure 3E).

**Figure 4:**
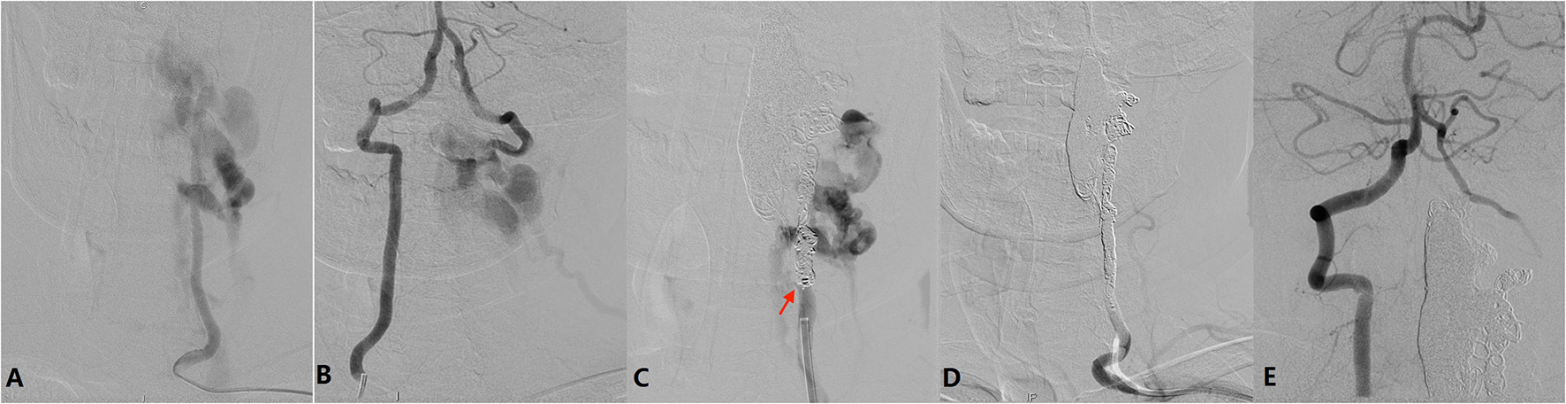
**(A)** Left vertebral angiography showing a large AVF located in the left C2-C6 level of the vertebral artery (VA). The fistula is fed by the proximal antegrade flow of the left VA. **(B)** Right vertebral angiography showing the AVF is fed by distal retrograde flow of right VA. **(C)** After occlusion of the venous plexus and distal VA, lower orifice (arrow) can be identified. **(D)** After endovascular embolization, left vertebral angiography showing the AVF together with left VA is occluded. **(E)** Right vertebral angiography showing a well preserved left posterior inferior cerebellar artery and null retrograde flow into the AVF.

Diagnostic angiography was performed via the right femoral artery. The aortic was extremely tortuous. Angiography showed a large AVF with multiple fistula orifices located in the left C2-C6 level of the vertebral artery (VA) (Figure 4A). The fistulae were fed by the proximal antegrade flow of left VA and distal retrograde flow of right VA (Figure 4B), and it was eventually drained to the external jugular vein through the vertebral and paravertebral venous plexus.

### Endovascular Treatment

Considering the persistent symptom being related to AVF, endovascular treatment was indicated. The procedure was conducted via a bilateral radial artery approach. Two Echelon-10 microcatheters (Medtronic Co. Minneapolis, USA) were placed in the biggest venous in C2-C4 via the upper fistula, and then six coils (Medtronic Co. Minneapolis, USA) were detached followed by injection of 6 ml Onyx 18 glue (Medtronic Co. Minneapolis, USA). However, the AVF was still existing after proximal VA angiography, indicating other fistulas also should be occluded. Then 3 more coils were placed in the V4 segment of the left VA to block the retrograde flow from the right VA. After that, 21 coils plus onyx 18 glue were injected into the C2-C6 segment of the left VA. After coil embolization step by step, the lower fistula orifice could be identified (Figure 4C). Finally, the AVF fistulas together with the left VA was occluded (Figure 4D). Angiography through the right VA showed a well preserved left posterior inferior cerebellar artery and null retrograde flow into the AVF (Figure 4E).

### Postoperative Course and Follow Up

The patient reported an improvement of neck pain and experienced no further neurologic deficits. Dramatically, the symptoms of tinnitus that bothered the patients for more than 30 years also improved significantly after the operation. Follow-up angiography performed 6 months later confirmed no recurrence of the AVF. And the patient was still free of symptoms till the latest follow-up.

### Genetic Detection

The proband genome DNA was extracted from venous blood using a Qiagen Mini Blood kit (Qiagen, Hilden, Germany) during follow-up. Whole-exome sequencing was performed using next-generation sequencing (NGS) and sanger sequencing was used to validate the results.

Whole-exome sequencing identified a novel germline heterozygous point nonsense mutation, c.G397T(p.E133X), in the NF1(NM_000267) gene exon4. Database (https://gnomad.broadinstitute.org/). The stop codon mutation is predicted to be functionally deleterious by *in-silico* software tools. Our finding was further supported by the conservative analysis across species. Rare variants of interest were classified for pathogenicity using the American College of Medical Genetics and Genomics guidelines.

## Discussion

Spontaneous vertebral AVF is extremely rare and is associated with a variety of vascular dysplastic disorders such as NF-1, fibromuscular dysplasia, and Marfan's syndrome (4). A series of cases of AVF associated with NF-1 have been published during the past decades and most cases were vertebral AVF (5), among which the female accounted for the majority. In these published studies, the primary symptoms were progressive neck pain or limb numbness/weakness, which might be attributed to radiculopathy of the nerve roots or the compression of the spinal cord. However, in our case, the patient also complained of a long history of tinnitus which was greatly improved after the AVF being cured. We speculate that tinnitus may be related to the high level (C1, C2) and high blood flow of the AVF, and the inner pressure of jugular vein might be increased which will result in reflux obstacle of cochlear lymph or blood flow.

The pathogenesis of AVF in dysplastic disorders is still unclear. Dysplasia of vascular smooth muscle was supposed to be a mechanism, which would result in vasculopathy, aneurysm formation, leakage, and rupture into the adjacent vein (6). The lack of neurofibromin, which is expressed by the NF-1 gene, might promote the pathological process (7). Neurofibromin functions in part as a negative regulator of the p21 Ras proto-oncogene, the loss of neurofibromin expression results in increased mitogenic signaling and thus increased cell growth, which in turn facilitates tumor formation (8). For AVF associated with NF1 cases, whether vertebral AVF in NF-1 is congenital or NF-1 disease progression hasn't been clarified. Previous literature proposed two possible mechanisms, one theory was that NF-1 sometimes presented as abnormalities of connective tissue which resulted in vessel vulnerability and friability, finally forming an AVF, the other theory was that AVF in NF-1 was congenital with a manifestation of mesodermal dysplasia. In some literature, it has been hypothesized that the loss of neurofibromin expression in endothelial cells may cause vascular smooth-muscle cells to dysplasia (8). For the formation process of AVF and aneurysm, Roth et al. proposed that aneurysm rupture into an adjacent vein may produce a pathological fistula, or a high-flow fistula may secondarily lead to aneurysm formation and eventual rupture (9).

In our case, the preoperative CTA images showed that there were multiple fistula orifices between left VA and paravertebral venous plexus, just like the schematic diagram in Figure 3E. This point was proved during the endovascular treatment. After occlusion of the upper VA from C4 to C2 segment, the AVF could still be clearly shown when contrast injection from the proximal VA, which means there still be some other fistula orifices in the proximal part of VA. Multiple fistula orifices indicate that the AVFs in NF1 might be congenital, which supports the hypothesis of the second mechanism. As in the first mechanism, AVF is easier to form a single fistula. This is because once the fistula is formed, the high-pressure arterial blood will find an outlet just like flood discharge, which will reduce the pressure of arterial wall in other parts, and once the fistula is formed, the pressure in the vein will increase, which will make it difficult to form another fistula. Furthermore, the VA is surrounded by the perivertebral venous plexus in the V2 segment anatomically, both of which were derived from mesoderm. We speculate that mesodermal dysplasia might result in the persistent orifices between VA and paravertebral venous, forming AVFs (10). By further whole-exome sequencing, a novel germline heterozygous point nonsense mutation was found in NF1(NM_000267) gene exon4, which led to the termination of the 133rd glutamic acid (Glu) in transcriptional level. The novel stop codon mutation is predicted to be functionally deleterious. Although there is no direct evidence, we highly suspect that the novel mutation might bring new clinical manifestations not reported in the literature to patients with NF-1, and AVF may be such a clinical manifestation. In the future, further more NF-1 patients with AVF and their families need to be investigated in order to confirm that the mutation is the root cause of NF-1 patients with AVF. And more basic research was also needed to establish a potentially direct link between the tendency to aggregate and the genomic code.

The scoliosis of this patient may be related to the imbalance of bilateral erector spinal muscle strength caused by AVF long-term compressing the cervical spinal cord and the syringomyelia. Combined with the long term of tinnitus, scoliosis, multiple orifices and the result of whole-exome sequencing, we preferred the AVF associated with NF-1 more likely being a congenital disease rather than a secondary one resulted in vessel vulnerability and friability.

AVF can be easily diagnosed by angiography, and two treatment strategies were reported in the literature, surgery, and embolization, both of which resulted in a good outcome (11). Referring to our case, as there were multiple fistula orifices and the AVF extended from C2 to C6 segment of the vertebra, there was a big challenge in hemorrhagic control and long segment exposure of the VA if we choose open surgery. Endovascular embolization is a good option with minimally invasive and completely occlusion of the fistula orifices for this patient. However, one disadvantage of this treatment is the sacrifice of the VA, meaning that the ipsilateral vertebral artery that give rise to the multiple arterial feeders is no longer functional for the normal vertebrobasilar circulation. Furthermore, multiple use of coils and glues in this patient also resulted in a high expenditure. An alternative method is to cover all fistulas with long segment covered stent. However, there is no such long covered stent available, and a long-term antiplatelet therapy is another disadvantage of using covered stent.

## Conclusion

Our reported case of vertebral AVF associated with NF1 showed multiple fistula orifices in the vertebral artery and a novel stop codon mutation in the NF1(NM_000267) gene exon4, which indicates that this rarely reported deformity might be a congenital disease with mesodermal dysplasia. With endovascular embolization, the AVF could be thoroughly occluded, and the patient can obtain a good outcome.

## Ethics Statement

The studies involving human participants were reviewed and approved by Institutional Review Board of Peking University First Hospital, Peking University. The patients/participants provided their written informed consent to participate in this study. Written informed consent was obtained from the individual(s) for the publication of any potentially identifiable images or data included in this article.

## Author Contributions

YW and CY wrote the report. SS and YZ care for the patient. JZ and HD confirmed the diagnosis and performed the treatment. Written consent to publish was obtained from the patient. All authors contributed to the article and approved the submitted version.

## Conflict of Interest

The authors declare that the research was conducted in the absence of any commercial or financial relationships that could be construed as a potential conflict of interest.

## Abbreviations

AVF: arteriovenous fistula
NF-1: neurofibromatosis type 1
MRI: magnetic resonance imaging
CTA: computed tomography angiography
VA: vertebral artery
